# A simple interpretation of undirected edges in essential graphs is wrong

**DOI:** 10.1371/journal.pone.0249415

**Published:** 2021-04-08

**Authors:** Erich Kummerfeld

**Affiliations:** Institute for Health Informatics, University of Minnesota, Minneapolis, Minnesota, United States of America; Queen Mary University of London, UNITED KINGDOM

## Abstract

Artificial intelligence for causal discovery frequently uses Markov equivalence classes of directed acyclic graphs, graphically represented as *essential graphs*, as a way of representing uncertainty in causal directionality. There has been confusion regarding how to interpret undirected edges in essential graphs, however. In particular, experts and non-experts both have difficulty quantifying the likelihood of uncertain causal arrows being pointed in one direction or another. A simple interpretation of undirected edges treats them as having equal odds of being oriented in either direction, but I show in this paper that any agent interpreting undirected edges in this simple way can be Dutch booked. In other words, I can construct a set of bets that appears rational for the users of the simple interpretation to accept, but for which in all possible outcomes they lose money. I put forward another interpretation, prove this interpretation leads to a bet-taking strategy that is sufficient to avoid all Dutch books of this kind, and conjecture that this strategy is also necessary for avoiding such Dutch books. Finally, I demonstrate that undirected edges that are more likely to be oriented in one direction than the other are common in graphs with 4 nodes and 3 edges.

## Introduction

Directed acyclic graphs (DAGs) are becoming increasingly important in fields such as epidemiology, biostatistics, operations research, and others as a tool for causal modeling and causal inference [1–7]. Often such graphs are drawn from background knowledge or expert opinion, but there is a substantial literature on statistical artificial intelligence methods that can identify one or more plausible causal models [8–12]. There is growing interest in using methods from this field, termed “causal discovery”, but this is hindered by confusion regarding how to interpret their output.

In this paper I shed some light on the interpretation of one of the most common types of output produced by causal discovery algorithms, the *essential graph*. Specifically, I provide a Dutch book argument that a simple interpretation of undirected edges in essential graphs is not rational. I also provide another theorem proving that a different interpretation can be used as the basis for a betting strategy that is not vulnerable to the same kind of Dutch book. I conjecture that the same betting strategy is also necessary to avoid being Dutch booked, but do not provide a proof for the conjecture.

### Background

Causal graphical modeling stems largely from [1, 8]. All of the important concepts used in this paper are covered in, or were introduced in, those publications. Those works make heavy use of statistical notions such as conditional independence, however such notions are not required for the theorems or proofs in this paper. In fact, the reader is required only to understand some limited terminology pertaining to directed graphical models, as well as rudimentary probability theory. The necessary background in graphical models is covered quickly in the Definitions section, while the reader’s knowledge of rudimentary probability theory is assumed.

### Importance

Causal discovery algorithms are increasingly being used on real world data sets to solve real world problems, e.g. [13–20]. It is rare for the result of such analyses to be a single DAG, so these papers commonly report graphs containing one or more undirected edges. These graphs are thus not DAGs, but more complex graph types such as essential graphs or mixed ancestral graphs [8]. A simple understanding of undirected edges in essential graphs is widely held by those using these methods, namely that “there is an arrow from one of these variables to the other, but it could point in either direction”. The more precise implications, however, are often not understood. Misunderstandings about such details are not a significant problem in and of themselves, however they can result in more serious errors, as this paper will show. When agents are trying to estimate precise quantities, such as the probability that an undirected edge in an essential graph has a particular directionality in the underlying DAG, these details become important.

Such estimates are becoming more common as researchers attempt to identify the best practices for using causal discovery algorithms. Methods such as resampling have begun to be evaluated as, and used as, tools for estimating confidence in discovered graphs, e.g. [15, 19, 21]. Specifically, these methods have been used to estimate the probability that the causal discovery algorithm has correctly identified particular edges. The presence of undirected edges in the discovered graphs produces a dilemma for users of this procedure, however. For example, let *X* and *Y* be a pair of variables in an analyzed data set, such that in 60% of the resampled data sets there is an undirected edge *X*—*Y* and in 40% there is the edge *X* → *Y*. What type of edge between *X* and *Y* should the user have the most confidence in? If we simply take the edge orientation that is most common, then it appears that the undirected edge is most plausible. If, however, we consider the undirected edges to be half directed in either way, then we infer that *X* → *Y* has 70% probability from the resampling analysis, and *X* → *Y* is most plausible. Undirected edges in essential graphs are not defined to have equal probability of being oriented in either direction, however such an interpretation may be tempting due to its simplicity or computational convenience. I argue here that rational agents should avoid this interpretation when possible, and offer another solution to this problem.

### Outline

In the Definitions section I define the terms to be used throughout the rest of the paper, and in the section following that I present the Dutch book argument against the equal orientation probabilities interpretation. In the section “Avoiding Dutch books” I prove that a different betting strategy is immune to such Dutch book arguments. In the Conjecture section I conjecture that the proposed betting strategy is also necessary for avoiding such Dutch books. In the section “Prevalence of imbalanced undirected edges” I analyze the specific case of graphs with 4 nodes and 3 edges, and evaluate the prevalence of undirected edges that are more likely to be oriented in one direction than the other. In the Discussion section I discuss the implications of these arguments. Finally, I summarize and conclude the paper in the Conclusion section.

## Definitions

This section is provided both as a very brief, rapid, and formal introduction to the topic and as a reference for the later parts of the paper. Many of these concepts have been defined in multiple different ways elsewhere, but the definitions provided here are largely equivalent, and are more suited to the purposes of this paper. Aside from the definition of Dutch book provided at the end, readers who are already familiar with the concepts and statistical properties of directed acyclic graphs, in particular the importance and meaning of uncovered colliders, may want to skip this section. Other readers may want to read it selectively to remind or refresh themselves on the material.

A *node* is a visual representation of a variable. The terms “node” and “variable” may be used interchangeably in this paper.

Like nodes and variables, *edges* can be both visual and abstract objects. Visually, edges are lines connecting nodes, sometimes augmented with arrowheads or other symbols along their length or at one or both endpoints. Abstractly, edges represent relationships between variables.

Two nodes are *directly connected* if there is an edge connecting them.

An *arrow* is visually an edge with an arrowhead at one end and an arrowtail at the other, or abstractly a representation of an asymmetric relationship between two variables.

An *undirected edge* is an edge that is not augmented with any arrowheads.

**Fig 1:**
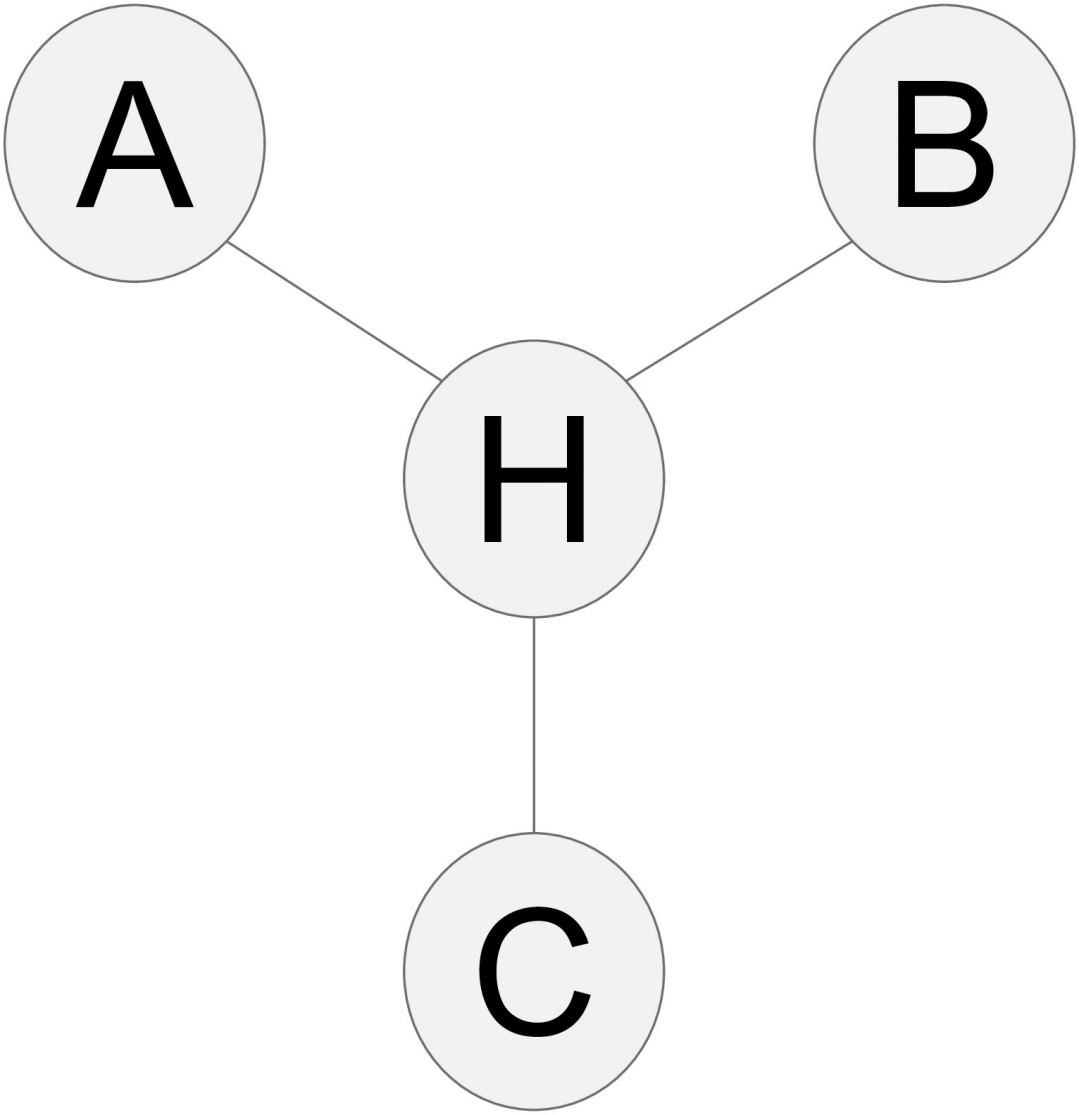
Essential graph *G* of variables A, B, C, and H. H is an undirected hub connecting the other three variables.

**Fig 2:**
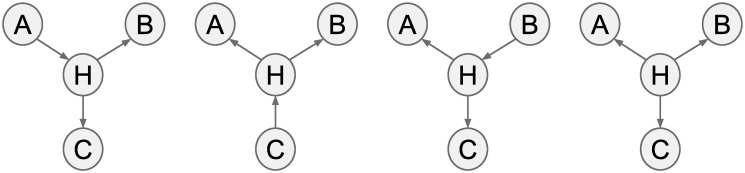
Visualizations of the four DAGs in *G*. H never has more than one incoming arrow.

A *graph* is a pair, 〈Φ, Ψ〉, where Φ is a set of nodes and Ψ is a (possibly empty) set of edges among the nodes in Φ. For any graph *G*, let Φ_*G*_ refer to the set of *G*’s nodes and Ψ_*G*_ refer to the set of *G*’s edges. Note that given the dual roles of all the terms used to define “graph”, it can be both a visual and an abstract object. Figs 1 and 2 contain examples of graphs.

An *undirected graph* is a graph containing only undirected edges. Fig 1 contains an example of an undirected graph.

A *directed graph* is a graph containing only directed edges. Fig 2 contains examples of directed graphs.

A *partially directed graph* is a graph that contains only directed and undirected edges.

Note that there are many other kinds of edges that partially directed graphs exclude, such as bidirected edges (edges with arrowheads at both ends), and edges augmented in other ways such as colors, dashed lines, or other kinds of symbols at the endpoints. Unlike graphs that might contain those more enriched edge types, partially directed graphs only permit two types of edges: directed and undirected. Directed graphs and undirected graphs are special types of partially directed graphs.

The *skeleton* of a graph is the set of directly connected pairs of variables in the graph. Two graphs can have the same skeleton if the same nodes are directly connected, even though the edges between those nodes may be different. More formally, graph *G* = 〈Φ, Ψ〉 has skeleton *S*(*G*) = 〈Φ, Ψ′〉 where Ψ′ = {(*X*, *Y*)|*X* and *Y* are directly connected in *G*}, with (*X*, *Y*) being the unordered pair of nodes *X* and *Y*.

A *path* in a graph *G* = 〈Φ, Ψ〉 is an alternating sequence of nodes in Φ and edges in Ψ, such that each edge directly connects the node before it to the node after it in the sequence. Paths both start and end with nodes. Paths are typically described as being *from* one endpoint and *to* the other endpoint, e.g. “a path from A to B.” An example of a path is 〈*A*, *A* → *B*, *B*〉. For brevity, when stating a path I will drop the repeated characters from the edges, e.g. 〈*A*, →, *B*〉.

A *directed path* in a graph is a path such that all arrows point in the same direction, e.g. 〈*A*, →, *B*, →, *C*, →, *D*〉. Directed paths have one node with no arrows pointed into it on the path, called the *source*, and one node with no arrows pointing out of it on the path, called the *sink*. Directed paths are typically described as going from the source and to the sink.

A *directed acyclic graph* (DAG) is a directed graph that contains no directed paths with multiple instances of the same node. Intuitively, this means that one cannot return to the same node in a graph if one follows the arrows in the graph. Fig 2 provides examples of DAGs.

A *collider* is any 3-node path of the form 〈*A*, →, *B*, ←, *C*〉. The term collider may also be used to refer to the middle node of such a path, but never to the other two nodes. Any node which is not a collider on a path is sometimes called a *noncollider*.

An *uncovered collider* is a collider path such that the initial and terminal nodes are not directly connected in the graph that contains it. For example, let *G* be a graph, let A, B, and C be nodes in *G*, and let A → B, and B ← C be edges in *G*. If A and C are not directly connected, then the path 〈*A*, →, *B*, ←, *C*〉 is an *uncovered collider*, otherwise it is a *covered collider*.

A path *P* is *active*, or *open*, conditional on a set of nodes **C**, if none of the noncolliders along *P* are in **C**, and if every collider along *P* is either contained in **C** or has a descendent in **C**. Otherwise, the path is considered *inactive*, or *closed*, or *blocked*.

Nodes A and B are *d-separated by* ***C***, or *d-separated conditional on* ***C***, in a graph, if there is no active path between A and B conditional on **C**.

Two graphs are *Markov equivalent* if they entail precisely the same d-separation statements. Note that this is equivalent to having the same skeleton and uncovered colliders.

An *essential graph* is a partially directed graph representing a collection of Markov equivalent DAGs. Fig 1 provides an example of an essential graph. The term essential graph will be used to refer both to the partially directed graph and to the set of DAGs that it represents. As such, one may write that the DAG D is a member of, or within, an essential graph E.

A *pattern* is a complete Markov equivalence class of DAGs. An essential graph is not required to contain all graphs which are Markov equivalent to its members. Unlike patterns, essential graphs may be learned from observational data paired with background knowledge, or from experimental data, or from other forms of enriched data that allows the researcher to restrict the space of permissible DAGs. Since the pattern concept is a special case of the essential graph concept, the theorems in this paper that pertain to essential graphs naturally also apply to patterns.

A *Dutch book* can be made against an agent if there is a gamble that appears acceptable to the agent but for which a loss is guaranteed. In other words, the loss occurs regardless of the outcome of the random event upon which the gamble is made.

Dutch book arguments have historically been used to support the axioms of probability theory, by proving that (1) any person who does *not* gamble in accordance with these axioms can have a Dutch book made against them, while (2) any person who *does* gamble in accordance with these axioms is immune to Dutch books [22–25]. In this paper I use a similar argument form as previous Dutch book arguments, but applied to the interpretation of essential graphs rather than to the theory of probabilities.

## A dutch book argument against equal orientation probabilities

**Theorem 1**. *If an agent always assigns equal probability to both edge orientations of any undirected edge in any essential graph, then there are cases in which they can have a Dutch book made against them*.

*Proof*. Let there be a DAG *D* over nodes A, B, C, and H. Let *D* be in essential graph *G*. Let *G* be such that there are undirected edges from each of A, B, and C, to H, and no other edges. In other words, *G* is a single hub network, with H being the hub. Fig 1 contains a visualization of *G*.

Let Steve be an arbitrary scientist who assigns equal probability to both edge orientations of all undirected edges in essential graphs. I will offer Steve the following package of bets about the DAG *D*, in the context that we know it is a member of pattern *G*:

1. Steve wins $6 if *D* contains the edge A → H, otherwise Steve loses $4
2. Steve wins $6 if *D* contains the edge B → H, otherwise Steve loses $4
3. Steve wins $6 if *D* contains the edge C → H, otherwise Steve loses $4

Steve calculates his mean expected profit or loss, *E*, should he accept the first bet:

$$\begin{array}{c} E=P\left(\text{A}\to \text{H}\in \Psi_{D}|D\in G\right)\cdot \text{6}+P\left(\text{A}\to \text{H}\notin \Psi_{D}|D\in G\right)\cdot \text{-4} \end{array}$$

Since Steve believes *P*(A → H ∈ Ψ_*D*_|*D* ∈ *G*) = 0.5, he calculates that *E* = 0.5 ⋅ 6 + 0.5 ⋅ -4 = 1. As such, Steve believes that this bet will on average profit him $1.

Following the same reasoning with the second and third bets, Steve comes to the same conclusion: he expects each bet to profit him $1. He calculates his expected profit for the package of all 3 bets as the sum of the expected profits of the individual bets, yielding $3. Since Steve expects to make money on average, he believes it is rational to accept the package of bets.

Steve’s expectation is wrong, however, as he is in fact guaranteed to lose money upon accepting this package of bets.

To see why, note that *G* is an equivalence class of DAGs with 4 members. Fig 2 contains visualizations of all 4 DAGs in *G*. Note that in no member of *G* does H have more than one incoming edge. This is because: for any DAG that has the same skeleton as *G*, if H has 2 parents, then that would form an uncovered collider, implying different d-separation relationships among the variables than those found in *G*. This creates a dependence among the orientations of the edges in *G*: if we learn that A → H, for example, then we can infer H → B and H → C, since that is the only graph in the equivalence class that includes A → H.

As such, Steve can only win at most one of the 3 bets in the package, and is guaranteed to lose at least 2. The best case scenario for Steve is a net profit of $6 + $4 + $-4 = $-2, or a loss of $2. By accepting this package of bets, Steve is guaranteed to lose at least $2, for all possible *D* ∈ *G*. Thus we have made a Dutch book against Steve.

The main idea behind this Dutch book is to construct an essential graph where assigning equal probability to both edge orientations in all unoriented edges is not consistent with any assignment of probabilities to the DAGs in that essential graph. Not all essential graphs are like this, and assigning equal probabilities to edge orientations does not not enable the construction of Dutch books for all essential graphs. For example, if the essential graph in question is *A*—*B*, a graph with only two nodes and one undirected edge, then any assignment of probabilities to *A* → *B* and *B* → *A* that sums to 1 cannot be Dutch booked.

For essential graphs like *G* in Fig 1, however, no assignment of probabilities to the four DAGs in that equivalence class, shown in Fig 2, can produce equal edge orientation probabilities for all three unoriented edges. Once this incoherence is established, any number of Dutch books can be constructed.

*G* is not the only type of essential graph with this property, and further, this situation cannot be resolved by adding additional nodes that connect to the 4 nodes in *G* (although it can be resolved by adding or removing edges in *G*). Essential graphs like *G* for which equal edge orientation probabilities are incoherent are not rare.

## Avoiding dutch books

A more complicated strategy for accepting gambles about edge orientations is immune to Dutch books of this kind. Informally, instead of the agent assigning edge orientation probabilities to individual edges without considering their larger context, this strategy begins by considering the entire set of DAGs in the equivalence class, creating a probability distribution over this set of DAGs. Individual edge orientation probabilities can then be computed from the probabilities of the DAGs.

In order to both state and prove the theorem, we need a few more definitions. Let *I*_*O*_(*A*, *B*, *D*) be the indicator function for determining whether *A* → *B* is in DAG *D*:

$$\begin{array}{c} I_{O}\left(A,B,D\right)=\{\begin{array}{cc} 1 & A\to B\;\text{is}\;\text{in}\;\text{DAG}\;D\\ 0 & \text{otherwise} \end{array} \end{array}$$

Let an *edge orientation bet* (EOB) be a 5-tuple *C* = 〈*G*, *A*, *B*, *R*, *L*〉 encoding the following bet. There is an unoriented edge between variables *A* and *B* in an essential graph *G*, for which a DAG *D* ∈ *G* will be revealed. An agent who accepts the bet has a change of *R* dollars if *A* → *B* in *D*, and *L* dollars if instead *B* → *A* in *D*. Formally, the agent receives *δ*_*C*_(*D*) = *R* ⋅ *I*_*O*_(*A*, *B*, *D*) + *L* ⋅ (1 − *I*_*O*_(*A*, *B*, *D*)).

Let a *collection of edge orientation bets* (CEOB) be a nonempty set of EOBs that all refer to the same essential graph *G*, and for which the same DAG *D* ∈ *G* will eventually be revealed, but is initially unknown. An agent who accepts a CEOB accepts all EOBs within it, which may refer to different undirected edges in *G*.

**Theorem 2**. *If an agent’s credences correspond to a probability distribution* *P*_*G*_ *over all DAGs* *D* ∈ *G*, *and the agent only accepts a CEOB E if* ∑_*C*∈*E*_ ∑_*D*∈*G*_ *P*_*G*_(*D*) ⋅ *δ*_*C*_(*D*) ≥ 0, *then no CEOB can form a Dutch book against that agent*.

*Proof*. Let our agent’s credences correspond to a probability distribution *P*_*G*_ over all DAGs *D*∈*G*. Let this agent only accept a CEOB *E* if ∑_*C*∈*E*_ ∑_*D*∈*G*_ *P*_*G*_(*D*) ⋅ *δ*_*C*_(*D*) ≥ 0. Since the agent accepts no other CEOB, then any CEOB which might form a Dutch book against the agent must satisfy that condition. Let *E* be such a CEOB, so:

$$\begin{array}{c} \sum_{C\in E}\sum_{D\in G}P_{G}\left(D\right)\cdot \delta_{C}\left(D\right)\geq 0 \end{array}$$

Since both sums are finite, we can reorder them, giving:

$$\begin{array}{c} \sum_{D\in G}\sum_{C\in E}P_{G}\left(D\right)\cdot \delta_{C}\left(D\right)\geq 0 \end{array}$$

If ∀_*D*∈*G*_ ∑_*C*∈*E*_ *δ*_*C*_(*D*) = 0, then it follows immediately that ¬∀_*D*∈*G*_ ∑_*C*∈*E*_ *δ*_*C*_(*D*) < 0, i.e. *E* is not a Dutch book. So, let us assume instead: ∃_*D*∈*G*_ ∑_*C*∈*E*_ *δ*_*C*_(*D*) ≠ 0. Since the sum over *D* ∈ *G* is ≥ 0, and one element of this sum is non-zero, then at least one of the elements of the sum must be > 0. This means:

$$\begin{array}{c} \exists_{D\in G}\sum_{C\in E}P_{G}\left(D\right)\cdot \delta_{C}\left(D\right)>0 \end{array}$$

Since *P*_*G*_(*D*) does not depend on *C*, we can move it outside the sum. Since *P*_*G*_(*D*) is positive, we can multiply both sides by 1/*P*_*G*_(*D*) without changing the inequality, leaving us with:

$$\begin{array}{c} \exists_{D\in G}\sum_{C\in E}\delta_{C}\left(D\right)>0 \end{array}$$

Introducing two negations outside of this statement, and then passing the inner negation all the way through to the inequality, gives us the following:

$$\begin{array}{c} \neg \forall_{D\in G}\sum_{C\in E}\delta_{C}\left(D\right)\leq 0 \end{array}$$

We can immediately infer the weaker statement:

$$\begin{array}{c} \neg \forall_{D\in G}\sum_{C\in E}\delta_{C}\left(D\right)<0 \end{array}$$

This final line explicitly negates that *E* is a Dutch book, concluding the proof.

Put plainly, this theorem states that agents who assign a coherent probability distribution to the DAGs in *G*, and only accept a gamble when their expected value for that gamble is at least zero, cannot be Dutch booked when the gamble pertains strictly to outcomes of various edge orientations.

## Conjecture

Theorem 1 showed that it is possible to be Dutch booked when one’s beliefs about edge orientations don’t align with the set of DAGs in the essential graph, and theorem 2 showed that those Dutch books can be avoided by forming beliefs about the set of DAGs prior to forming beliefs about edge orientations. It remains to be shown that *only* those agents who form beliefs in accordance with theorem 2 can avoid those Dutch books.

Since we are now considering that agents may form probabilistic beliefs regarding individual edge orientations, rather than entire DAGs, we need to add some additional notation. Let *P*_*α*_(*A*, *B*) be agent *α*’s probabilistic beliefs about whether edge *A* → *B* will be in the revealed DAG *D* ∈ *G*. Let *δ*_*C*_(*A*, *B*) be the change in value to an agent who accepts bet *C* after it is found that the edge *A* → *B* is in the revealed DAG *D* ∈ *G*.

**Conjecture 1**. *Given an essential graph G*, *if an agent α has beliefs P*_*α*_(*A*, *B*) *that are not consistent with any P*_*G*_ *over all DAGs D* ∈ *G*, *i.e*. *if*

$$\begin{array}{c} \neg \exists_{P_{G}}\forall_{A,B}P_{\alpha }\left(A,B\right)=\sum_{D\in G}P_{G}\left(D\right)\cdot I_{O}\left(A,B,D\right) \end{array}$$

*then they can be Dutch booked with a CEOB*, *i.e*. *there exists a CEOB E s.t*.

$$\begin{array}{c} \forall_{D\in G}\sum_{C\in E}\delta_{C}\left(D\right)<0 \end{array}$$

*and*

$$\begin{array}{c} \sum_{C\in E}\left[P_{\alpha }\left(A,B\right)\cdot \delta_{C}\left(A,B\right)+\left(1-P_{\alpha }\left(A,B\right)\right)\cdot \delta_{C}\left(B,A\right)\right]\geq 0 \end{array}$$

Should this conjecture be proven, it would imply that the sufficient condition for avoiding CEOB Dutch books defined in Theorem 2 is also a necessary condition for avoiding CEOB Dutch books. I have attempted to prove this conjecture, but found progress difficult due at least in part to there being many different ways in which some *P*_*α*_ may not be consistent with any *P*_*G*_, and due to the large space of possible CEOBs which one might try exploit to construct a Dutch book. Different such *P*_*α*_’s might require very different CEOBs in order to be Dutch booked, and I found it difficult to characterize all such interactions.

## Prevalence of imbalanced undirected edges

It is clear that there exist undirected edges which, assuming a uniform distribution over the DAGs in the essential graph, do not have a 50% chance of being oriented in either direction. Such edges might be rare, though. How likely is it that any particular undirected edge we encounter is a 50% edge versus an edge biased towards one direction or the other?

The likelihood of 50% edges is greatly dependent on graph properties such as the ratio of edges to nodes in the graph. In completely connected patterns, for example, all undirected edges are 50% edges. Similarly, in very sparse graphs where all edges are isolated from each other, preventing paths of length 2 or greater from forming, all undirected edges are also 50% edges. The more interesting graphs are those in the middle ground, with enough edges to form longer paths but not to form large heavily connected subgraphs.

**Fig 3:**
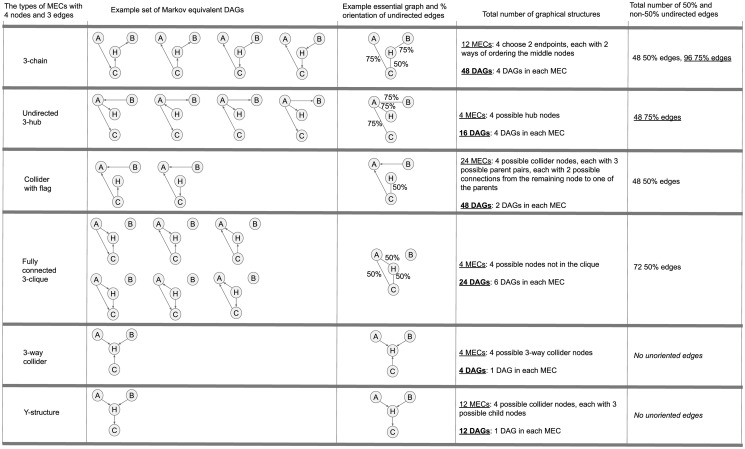
Visualization of examples of the 6 types of Markov Equivalence Classes (MECs), their corresponding essential graphs, the DAGs contained in those essential graphs, the total number of DAGs that fall into each type of essential graph, and the quantity of undirected edges with equal and unequal odds of being oriented in either direction, given a uniform distribution over the DAGs. In addition to undirected hub graphs, undirected chain graphs also contain undirected edges which are more likely to be oriented in one direction. With 4 nodes and 3 edges, other than 50% edges there are only 75% edges and completely directed edges. Overall, 46% of the undirected edges are 75% edges, indicating that non-50% edges are common among graphs with 4 nodes and 3 edges.

Fig 3 shows all 6 types of node-permutation equivalent patterns that have 4 nodes and 3 edges, and provides examples of each along with their corresponding set of DAGs. It indicates the likelihood of each undirected edge in the pattern of being directed in one direction or the other, and calculates the total number of DAGs that fall under some pattern of each node-permutation equivalent type. The number of total undirected edges produced from the DAGs in each type is shown, broken down by maximum % orientation in either direction.

There are a total of 152 DAGs with 4 variables and 3 edges. There are $(\begin{array}{c} 6\\ 3 \end{array})=20$ skeletons, each with 2^3^ = 8 ways of orienting the 3 edges, for a total of 160 directed graphs. Of these, 8 contain directed cycles (2 for each skeleton with 3 fully connected nodes), and are thus not DAGs. In total, (96 + 48)/(96 + 48 + 48 + 48 + 72) ≈ 46% of the unoriented edges are 75% oriented in one direction. While this example only concerns graphs with 4 edges and 3 nodes, it indicates that undirected edges strongly biased towards one direction may be common.

## Discussion

An essential graph consisting of a single undirected edge is safe from Dutch books no matter how the agent assigns probabilities to that edge’s orientations (assuming they still abide by the basic rules of probability, of course). While ignoring the inter-dependencies of orientations of undirected edges does not guarantee that an agent will be vulnerable to a Dutch book, it does leave open that possibility. The “50% either way for all unoriented edges” rule makes an agent vulnerable to Dutch books, but not for all essential graphs. It is still enough to be disatisfied, since an alternative is available.

These theorems have implications for at least a few real world statistical settings. First, a domain expert who has used a pattern-learning statistical procedure to estimate the Markov equivalence class of causal DAGs will often wonder how likely it is for the undirected edges to be oriented one way or another. It is unsatisfying, and perhaps even psychologically impossible, to have no credence whatsoever for these graph features. Treating the odds of two possible outcomes as a coin toss is simple and intuitive, when one lacks any information suggesting one is more likely than the other. It is thus plausible that numerous researchers who have used causal discovery software are vulnerable to a Dutch book. Exposure to the theorems contained in this paper might make these researchers more careful when interpreting undirected edges in patterns.

Second, there is some uncertainty regarding how to treat undirected edges in ensembles of essential graphs. One procedure is to, for each pair of variables, report the proportion of times each of the possible edge orientations occurred [15]. The theorems in this paper suggest, however, that we ought not to interpret undirected edges without considering their context, i.e. the essential graph that they occur within. This makes interpretation of the frequency of undirected edges in a table of edge orientation frequencies difficult, as such a table removes any such notion of graphical context of individual edges. It is possible that the count of undirected edge orientations is summing over what is actually a highly heterogeneous collection of edges, despite them all being undirected.

A third point follows from the other two. When considering how “correct” an ensemble of essential graphs is relative to a target DAG, there is some question as to how to treat the learned undirected edges. Without knowing the rest of the graph that these undirected edges are part of, many of those undirected edges may come from patterns in which they are much more likely to be oriented in one direction or the other, assuming a uniform distribution over the Markov equivalent DAGs.

## Conclusion

In this paper I’ve stated and proven two theorems about the interpretation of essential graphs. The first theorem states that a simple interpretation of undirected edges, namely giving equal credence to both orientations of all such edges, leads to a Dutch book. The second theorem states that a more complex interpretation, which takes the entire graph into account rather than attempting to interpret edges independently of each other, is immune to such a Dutch book. I conjectured but did not prove that any interpretation that is not consistent with this one will be vulnerable to a Dutch book. In other words, I proved that this interpretation is sufficient for avoiding a Dutch book, and conjectured that it is also necessary. Finally, this paper also demonstrates that undirected edges which are more likely to be oriented in one direction than another may be quite common.

This paper focuses on essential graphs, which are a simple and widespread graphical representation of Markov Equivalence Classes of DAGs, but essential graphs have representational limitations. For example, essential graphs cannot represent the possibility of an unmeasured common cause of two or more measured variables. Other types of graphs, such as Mixed Ancestral Graphs, are capable of representing these more complex graphical structures and their corresponding Markov Equivalence Classes. Future work should extend the ideas in this paper to these more expressive but more complicated graphs.

Theorem 2 demonstrates that considering the entire set of graphs in the Markov Equivalence Class can protect us from being Dutch booked, but this may be difficult to implement in practice. This difficulty is primarily computational: Markov Equivalence Classes can contain large numbers of DAGs. These sets can be so large that enumerating all their members, let alone reasoning from all of them simultaneously, is prohibitively computationally expensive. In many graphs, though, one would not actually need to consider the entire set of DAGs in order to determine safe credences about edge orientations. For example, a graph could have two completely disconnected subgraphs, in which case the edges in either subgraph would not be relevant to determining credences about the edge orientations of the other subgraph. Previous work has developed computationally efficient ways of estimating total causal effects for all DAGs in an equivalence class [26]. Similar methods should be explored in order to develop computationally efficient procedures for computing safe credences about edge orientation probabilities.
